# Supplementary figures and images for: Valve cells are crucial for efficient cardiac performance in Drosophila
Source: PLoS Genet. 2025 Mar 20;21(3):e1011613. doi: 10.1371/journal.pgen.1011613 (PMC11925464; doi:10.1371/journal.pgen.1011613)

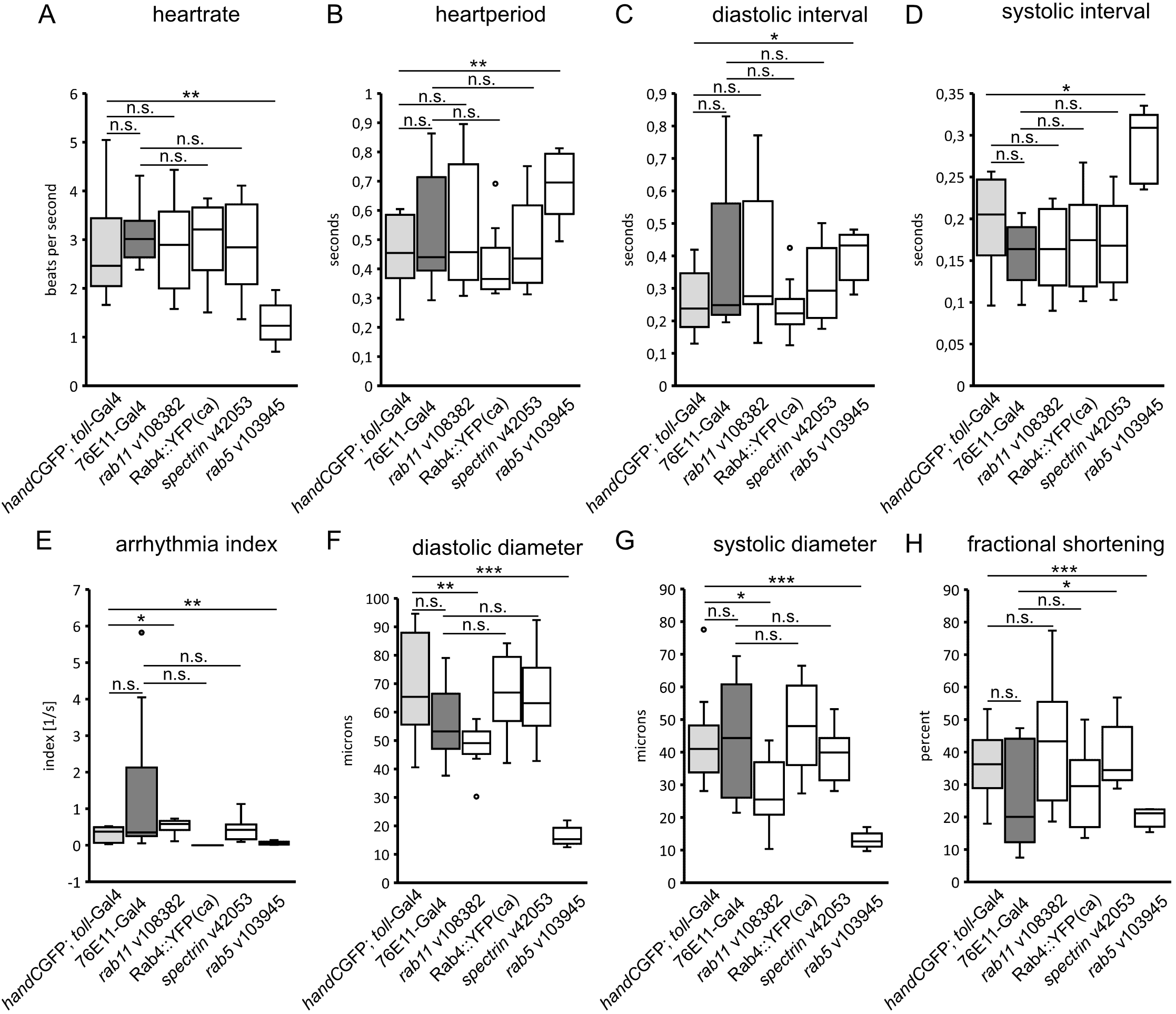

Supplement: S1 Fig — Knockdown of rab11 results in reduced diastolic and systolic diameters and an increased arrhythmia index. Ectopic expression of Rab4 increases the number of valvosomes and reduces their size, but cardiac parameter were unaffected. Knockdown of spectrin reduced valve cells deformation capability and luminal distance, as well as fractional shortening. Downregulation of rab5 negatively affected cell and valvosomal size, as well as number of valvosomes. In addition, heartrate, diastolic and systolic diameter and fractional shortening were significantly reduced. Cardiac malformation leads to reduced cardiac pumping efficiency and hemolymph distribution. N = 10 animals per genotype were investigated, for rab5 knockdown lines five animals, and data were analyzed using an unpaired two-tailed Student’s t-test *P < 0.05, **P < 0.01, ***P < 0.001. (TIFF) [file pgen.1011613.s001.tiff]
