# Supplementary material for: Valve cells are crucial for efficient cardiac performance in Drosophila
Source: PLoS Genet. 2025 Mar 20;21(3):e1011613. doi: 10.1371/journal.pgen.1011613 (PMC11925464; doi:10.1371/journal.pgen.1011613)
Supplement: Attachment — Submitted filename: Rebuttal Letter_PLOS GENETICS.pdf [file pgen.1011613.s006.pdf]

Dear Editor

We are grateful to all three reviewers for their constructive comments on our work and for the insightful suggestions. As explained in more detail below, we have addressed all points raised by the reviewers, and most of them have led to numerous improvements in the manuscript. In this letter, we have used a color code to explain our actions and changes (black = reviewers comment, red = author's response, blue = actions taken). We have uploaded a formatted PDF version of this point-by-point response as supplementary information.

Reviewer 1 Comments for the Author:

Reviewer's comment: The study provides a comprehensive overview of hemolymph flow and cardiac valve function in Drosophila but would benefit from a discussion of why the authors chose to study these particular genes of interest.

Author's reply: We thank the Reviewer and apologise for any misconceptions. The genes we have chosen for this recent work have been identified by us as relevant components of valve formation. We published this in 2022 (Meyer et al. 2022). We identified several critical genes, which, upon down-regulation or expression as constitutively active variants, induce different types of malformations in valve cells without affecting neighbouring cardiac cells. As a result, we shed light on the biogenesis and maintenance of the valve cell and critical genes. However, physiological consequences of valve malformation were elusive. Thus, we chose particular genes previously identified by us to generate reliable phenotypes for physiological analysis.

Reviewer's comment: Fig. 1 - This figure legend indicates that manipulating Rab5, which facilitates migration of valve cells to their final location in the lumen via endocytosis, and Rab4/11, which regulate the recycling of endosomes responsible for valve cell transport, leads to smaller valve cells and absent valvosomes. These differences should be quantified and included in this figure.

Although the schematics shown in D'-H' could be useful, they don't correspond to what is shown in the actual micrographs D-H.

The authors should elaborate on the methodology used in Figure 1. While the phenotype is clearly depicted, the text currently offers only a superficial reference to a previous paper. More detailed methods, quantifications and explanations should be included for the reader of the current article.

Author's reply: We agree with the Reviewer that the manuscript might benefit from a more detailed explanation of the previous results, which we will incorporate into the revised version. In the same line, we hope that it becomes clear, that schematics should represent general graphic illustrations of the phenotypes previously found and analysed in this study. They are for easy, clear and fast perception of differences and

characteristics of the phenotypes rather than representing a redrawn scheme of microscopic images.

Actions taken: We now included a more detailed description and reference to our previous results in the main manuscript according to the Reviewer's suggestion.

Reviewer's comment: Fig. 2,3,4,6,8, S1 – How the authors used the two different drivers (76E11-Gal4 and Toll-gal4) is unclear. The authors should clearly indicate which driver was used to modulate expression when crossed to the different rab and spectrin lines. Ideally, each driver should be crossed to all of the KD or OE lines and the results shown for each driver set. This would ensure that results are consistent regardless of driver, i.e. in differing genetic backgrounds. Were additional KD lines tested for each gene?

Author's reply: We apologize for missing/ unclear information regarding the use of the drivers. Both drivers are specifically active in 3rd instar larval valve cells and were used according to previous studies (Meyer et al. 2022, Development. 2022 Oct 1;149(19):dev200701. doi: 10.1242/dev.200701.; Meyer et al. 2023, Genesis. 2023 Mar;61(1-2):e23506. doi: 10.1002/dvg.23506.) and for technical reasons in order to ensure phenotype reproducibility for our physiological experiments in this study.

Nevertheless, we performed internal control experiments for each group and between drivers as shown in the manuscript. In addition, knock-downs showed similar results and phenotypes, regardless of the driver used. The Reviewer is kindly referred to the attached example figure showing similar phenotypes upon knockdown of *rab5* or *rab11* independent of the driver line used.

In addition, we tested two different RNAi-mediating overexpression lines for each gene and found similar phenotypes concerning morphology and physiology; a summary may be found in our previous publication (Meyer et al., 2022).

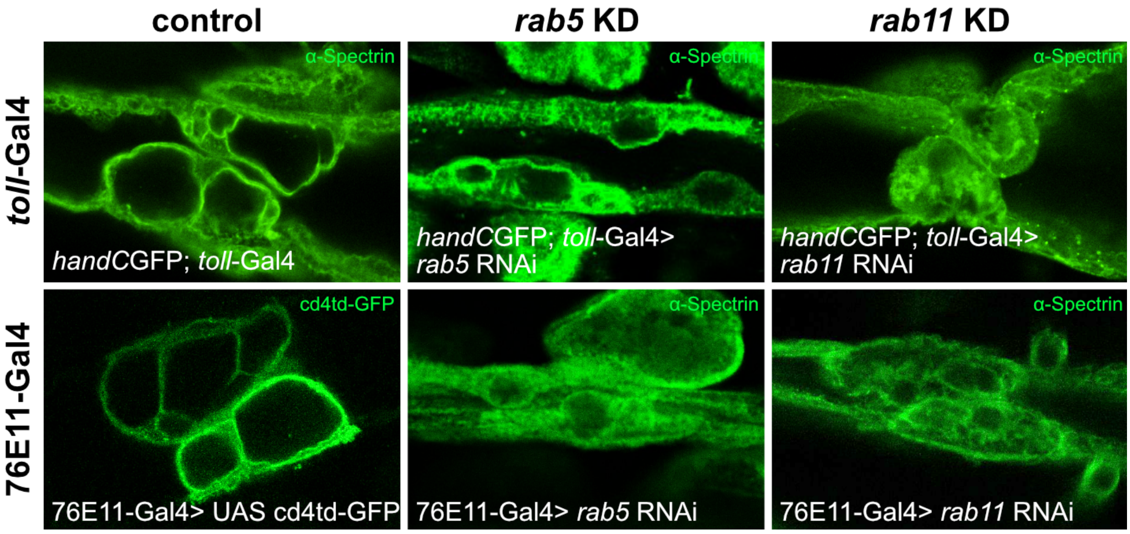

Actions taken: We updated all figures of the manuscript according to the Reviewer's suggestion and clearly indicated which drivers were used in each experiment.

Reviewer's comment: The expression patterns for each driver line should be documented and/or cited. In particular it is not clear where 76E11-Gal4 is expressed, the available literature indicates it is a neuronal driver, how does that fit with this experiment on valves? Is the Toll-Gal4 expression exclusively in valves and no other tissues? Was the extent of KD quantified?

Author's reply:

We agree, the expression pattern of the toll-Gal4 and the 76E11-Gal line is very important to show. We did this already and published our results on the the expression patterns in the following papers: Toll-Gal4 in Lammers et al 2017, Formation and function of intracardiac valve cells in the Drosophila heart. The Journal of Experimental Biology, 220, 1852–1863. 76E11-Gal4 (Tail-up-Gal4) in Meyer et al 2023 in Tailup expression in Drosophila larval and adult cardiac valve cells. genesis, 61(1–2), e23506. 76E11 is also described in Morin-Poulard et al. 2022.

The extend of knockdown was quantified by us in previous studies and was reproducible by two independent lines per genotype (Meyer et al. 2022). Furthermore, the efficiency of the RNAi lines has been validated in larval fat bodies for ***rab5*** (Jacomin et al. 2016) or neurons for ***rab11*** (Lin et al. 2020), resulting in knockdown efficiencies up to **85%**.

Actions taken: We have cited the relevant papers for driver expression patterns in the Material & Methods section of the manuscript.

Reviewer's comment: For data in Fig. 2 & 3, how was the pixel intensity data normalized to allow comparisons between genotypes? For example, peak intensities for controls in Fig. 3 D & E are ~7 to 8, however, peak intensities for Rab11 KD and spectrin KD (F & H) are higher at ~ 10 to 11; yet the quantification shown in J indicates significant reductions in flies with Rab11 or spectrin KD compared to controls.

Author's reply: We thank the Reviewer for pointing out the lack of clarity for some of our measurements. Pixel intensity analysis was done as described in Choma et al. 2011 and Drechsler et al. 2013. In brief, pixel intensity within the ROI was measured before dye injection and set as background. This background intensity was subtracted from all the following measured intensities within the ROI during dye angiography analysis. Background intensity, which is mainly influenced by the individual darkening of the cuticle, was calculated for each animal individually before analysis.

Figures 3 D & E and 3F &H illustrate dye oscillograms from single individuals with a squared-shaped ROI located directly anterior to the valves to highlight valve closing capabilities. As a result time varying intensities of single dye packages passing the ROI are recorded. In fact, dye packages extend within the aorta in the anterior direction upon heart beating and streaming anteriorly. ROI will cover only time-

varying parts of the dye packages. In contrast, Figure 3 J highlights the dye intensity of the total dye package, where the complete area of the dye packages is analysed. Thus, a direct comparison between these measurements is not possible.

Actions taken: We edited the related parts in the Material & Methods section of the manuscript according to the Reviewer's suggestion. In addition, we updated Figure 3 with a color-coded scheme to further clarify the intensity curves of oscillograms and dye bulk package analysis.

Reviewer's comment: The conclusions in Figure 6 are inconsistent with Supplemental Figure 1, where diastolic diameter is reduced in Rab11 and Rab5 KD models.

Author's reply: We understand the idea of the Reviewer of linking results i.e. to strengthen a hypothesis. However, in this case it was not our aim to compare these data, since they were based on completely different, technically diverse and independent experiments. For instance, analysis of heart myofiber structure (Figure 6) was done after complete dissection of animals in an $MgCl_2$ enriched buffer, while for SOHA analysis, animals were bathed in artificial hemolymph, and the least amount of viscera were removed.

Reviewer's comment: More detail should be provided as to how the densities and myofibril orientation were obtained in Fig. 6. Did the authors analyze specific ROIs within the heart, if so where, how many / heart? Show examples of the myofibrillar organization in the KD and OE lines.

Author's reply: We appreciate the Reviewer's query on the myofibril analysis and are deeply sorry for the missing information. Myofibril densities and orientations were measured in a single 100x50 µm ROI in the abdominal segment 5 (A5) between pairs of functional ostia to exclude a potential effect of the ostia-specific muscle fibre organization.

Actions taken: We have updated the related parts of the Material and Method-section respectively. In addition, we designed a new Figure 6 and added images for the KD and OE lines, as suggested by the Reviewer.

Reviewer's comment: For data presented in all Graphs (except Fig. 5G, where only two groups are being compared) the appropriate statistical test would be a one-way ANOVA.

Author's reply: We thank the Reviewer for providing valuable notes on the statistical analysis. We agree that a one-way ANOVA is the best choice for statistical analysis when comparing more than two groups with each other. However, we do not intend to do so, as we would like to highlight individual differences between groups i.e. different degrees of statistical significance, to gain more detailed insights into

physiological consequences. Nevertheless, we performed ANOVA analysis and Kruskal-Wallis test where applicable and found similiar results and P-values for our experiments. A brief summary may be find in the table below:

Table 1. Statistical analysis of all groups within a single data set and respective statistical analysis applied.

Figure	Statistical Test	P-Value	significant Difference between Groups?
Fig.2 J Dye Angiography	Kruskal-Wallis test	<0,000001	yes
Fig.3 J Dye Bulk Intensity	one-way ANOVA	0,000028	yes
Fig.3 K Hemo. Velocity	Kruskal-Wallis test	0,307163	no
Fig.4 Q Luminal Distance	one-way ANOVA	0,000082	yes
Fig.4 R Opening Time	one-way ANOVA	0,123943	no
Fig.6 G Heart Area	one-way ANOVA	0,261285	no
Fig.6 H Myo Density	one-way ANOVA	0,172837	no
Fig.6 I Directionality	one-way ANOVA	0,25092	no
Fig.8 B Track Distance	Kruskal-Wallis test	<0,000001	yes
Fig.8 C Confinement Ratio	Kruskal-Wallis test	<0,000001	yes
Fig.8 D Mean Speed	Kruskal-Wallis test	<0,000001	yes
Fig.8 E Displacement	Kruskal-Wallis test	<0,000001	yes

Reviewer's comment: Given the large SEMs in most of the graphs there appears to be a very large amount of variability in these samples (including even in the controls), making some of the indicated significant differences rather questionable and indicated significant differences will likely change when the correct statistical tests are applied. Graphs should show all data points so the reader can get a feel for this variability. Because physiological data can be variable this submission would benefit from higher Ns for most of the analyses.

Author's reply: We are aware of the common problem of variability in physiological data. Our experiments were inspired by the very few studies on hemolymph dynamics and dye angiography experiments in insects, as referred in the material & methods section. These studies had sample sizes of ~5 animals per group (see Choma et al., 2011), which we also found ambitious and thus, and thus decided to double the sample size. We agree with the Reviewer that graphs should include all data points for the reader to get an impression on variability

Actions taken: We have changed all bar graphs into boxplots according to the Reviewer's suggestion and added data points to illustrate variability of the data to the reader.

Reviewer's comment: Fly stock codes do not need to be included in the figures themselves. These should be specified in a separate table in Methods or supplemental data. This will make interpretation of the genotypes in the graphs a little easier.

Actions taken: We have changed all figures according to the reviewer's suggestion and added fly stock codes in the material & methods section.

Reviewer's comment: The specific driver line crossed to each rab and spectrin line should be indicated. It is not clear from the graphs that Rab4 is an overexpression line.

Actions taken: We have changed all figures according to the Reviewers suggestion.

Reviewer's comment: Figures 2 and 3 could potentially be reduced and combined to minimize redundancy (Fig. 3a for example does not provide more info than is available in Fig. 3b) and improve flow.

Author's reply: We appreciate the reviewer's suggestion of combining figures. However, our two figures show different types of analysis and regions of interests for measurements and evaluation, which may confuse the reader when combined. In addition, we do not exceed the journal's maximum figure number and would like to keep the figures as submitted.

Reviewer's comment: Fig. 4, It is unclear how the luminal distance was determined for valves that are not completely juxtaposed. It would be helpful to include a line showing where this distance is measured. In addition to control images shown in G-K, authors should include examples of at least one of the KD or OE lines.

Author's reply: We are sorry for an unclear description of the measurement process. Luminal distance was measured as the shortest distance between opposing cells (cytoplasmic border of valve cells) if viewed from inside the heart in a 90° angle. We agree with the Reviewer's opinion that an additional indication in the respective figure would be helpful and that additional example images of KD or OE lines are valuable.

Actions taken: We have updated and corrected Figure 4 according to the Reviewer's suggestion.

Reviewer's comment: Fig. 5, depicts an interesting analysis of blood flow velocities but why is only one gene of interest included in this analysis since all the genes tested seem to affect valves and flow in some way? It is unclear what is being shown in the red box in (B) and what is narrowing.

Author's reply: Our previous studies indicated, that the *rab11* knockdown leads to the most severe, but very specific, phenotype with valve cells completely lacking valvosomes and having a highly reduced cell size and deformation capability, see our previous paper (Meyer et al. 2022). Only the *rab5* knockdown leads to even more severe phenotypes, where valves cannot properly close the heart lumen and pump hemolymph (this study). Since the experiment is technically challenging and highly complex to execute, we focussed on *rab11* KD lines as the best candidate to analyse potential effects on hemolymph streaming properties. The red box in Fig. 5B indicates the position/ area of the heart lumen spanned by the intracardiac valves. Within this area, valves deform upon heart beating and create a narrowed heart lumen in the respective KD and OE lines.

Reviewer's comment: Fig. 6, the data for Rab5 appears inconsistent with an n=10 sample size based on the error bars. This discrepancy warrants further clarification.

Author's reply: We are deeply grateful to the Reviewer for critically evaluating the presented data and for identifying our mistake. We apologize for the typo in Figure 6.

Actions taken: We have updated and corrected Figure 6 according to the Reviewer's suggestion.

Reviewer's comment: Fig. 6, please provide more explanation of what is shown in A',B' and C', please show examples of the myofibrillar structure from the spectrin and Rab5 lines.

Author's reply: We appreciate the Reviewer's query on the myofibril analysis and are deeply sorry for the missing information. We edited and updated Figure 6 for more clarity. In brief, panel A-F show F-Actin staining of larval heart chambers and the position of the orthogonal section within the abdominal segment 5 (A5, orange line) for heart area analysis. The resulting cross-section of the heart is shown adjacent to the images in the orange boxes and was measured for the heart area. Panel A'-F' show colour-coded images of the myofibril orientation of the heart chamber. Myofibril densities and orientations were measured in a single 100x50 µm ROI in the abdominal segment 5 (A5, white dashed boxes in A'-F') between pairs of functional ostia to exclude a potential effect of the ostia-specific muscle fibre organization.

Actions taken: We edited and updated Figure 6 according to the Reviewer's suggestion.

Reviewer 2 Comments for the Author:

Reviewer's comment: In Figure 1 authors present a summary of valve phenotypes observed in a serie of identified previously mutants and different valve targeting GAL4 lines. It would be important to provide more information about the valve driver lines and specificity of their expression and explain the reason two different drivers are used to generate valve phenotypes in Fig. 1 but also in following experiments.

Author's reply: We appreciate the Reviewer's interest in driver lines and expression patterns. The expression pattern of the toll-Gal4 and the 76E11-Gal line is very important to show. We did this already and published our results on the the expression patterns in the following papers: Toll-Gal4 in Lammers et al 2017, Formation and function of intracardiac valve cells in the Drosophila heart. The Journal of Experimental Biology, 220, 1852–1863. 76E11-Gal4 (Tail-up-Gal4) in Meyer et al 2023 in Tailup expression in Drosophila larval and adult cardiac valve cells. genesis, 61(1–2), e23506. 76E11 is also described in Morin-Poulard et al. 2022.

In short, both drivers are exclusively active in valve cells of 3rd instar larvae and the different combination of drivers was used to generate reliable and reproducible valves phenotypes of previous studies. Both drivers induce identical phenotypes when used for knock-downs. The Reviewer is kindly referred to the adjacent image below, highlighting that the *rab5* KD and *rab11* KD phenotypes can be generated by the *toll* and the *76E11* enhancer line.

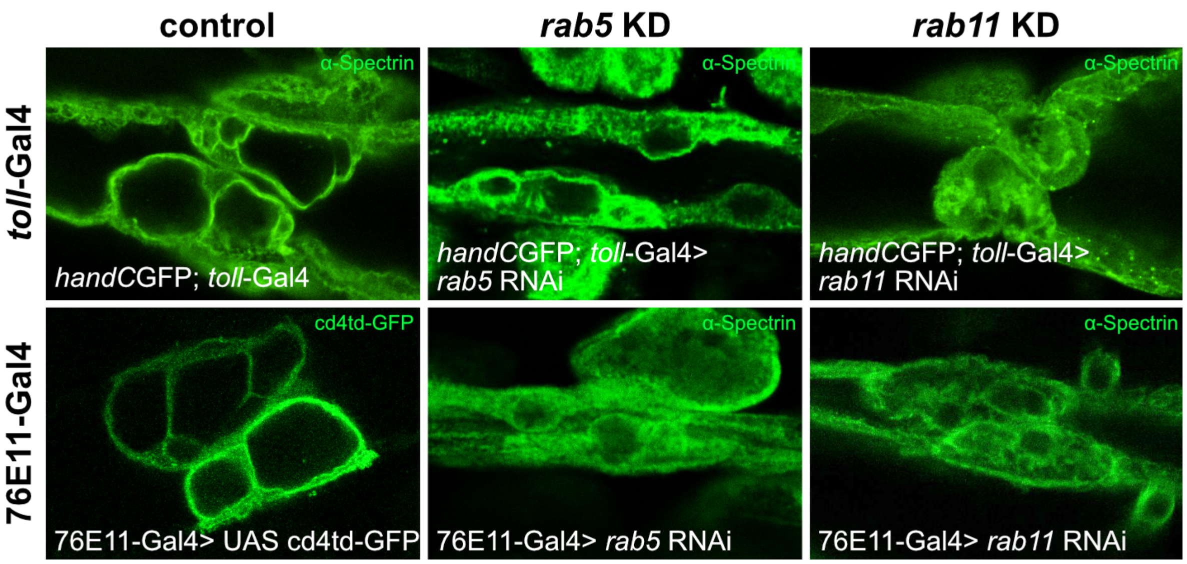

In addition, we performed internal controls within each group and between drivers (this study) and analyzed additional RNAi lines for each gene, which induce similiar phenotypes (Meyer et al., 2022).

Reviewer's comment: Both Rab11 and Rab 5 KD lead to a loss of valvosomes, however Rab5KD impact's on heart parameters is much more severe than that of Rab11KD. This needs to be discussed especially with respect to hemolymph velocity (Fig. 3K).

Author's reply: We thank the Reviewer for critical analysis of the *rab11* KD and *rab5* KD lines. In a previous study, we proposed *rab5* as a crucial factor for initial valvosome formation, while *rab11* is crucial for proper maturation and maintenance of the valvosomal compartment (Meyer et al., 2022). Upon depletion of *rab5* valves posses a cardiomyocyte appearance and are (in the most cases) unable to properly close the heart lumen. This is why hemolymph velocity could not be determined for *rab5* KD lines in Fig. 3K. Not fully functional valves may also influence the heart in general, leading to more severe heart parameters.

Actions taken: We added the relevant information to the manuscript's results section.

Reviewer 3 Comments for the Author:

Reviewer's comment: Statistics: change all bar graphs to box/whisker and show all data points; t-tests require normally distributed data - was this ensured? For non-normal data Wilcoxon tests are warranted.

Author's reply: We agree with the Reviewer that boxplots are more valuable for the reader when interpreting the data. In addition, adding single data points helps understanding variability within each data set.

Actions taken: We have changed all bar graphs to boxplots and added data points, as suggested by the Reviewer. In addition, we carefully re-analyzed data and applied nonparametric tests where necessary.

Reviewer's comment: Crawling Assay Interpretations: The link between reduced crawling and valve malfunction could be influenced by external factors not accounted for, such as genetic pleiotropy. Both controls show already differences between each other. I am not sure about the significance of this experiment.

Author's reply: We thank the Reviewer for this interesting statement. We agree that external factors may impact the crawling assay. However, we tried to diminish external factors by strictly regulating, i.e., the age of the larvae, time during the day for the crawling test, temperature, humidity, light and stress by letting larvae rest before imaging. Nevertheless, the Reviewer is right, and we constitute our experiment as a very first and straightforward assay, trying to link behaviour to valve malfunctions. Future studies, for example in adult flies, may shed more light on this fascinating topic. However, this will be an independent story on its own.
